# Procathepsin V Is Secreted in a TSH Regulated Manner from Human Thyroid Epithelial Cells and Is Accessible to an Activity-Based Probe

**DOI:** 10.3390/ijms21239140

**Published:** 2020-11-30

**Authors:** Alaa Al-Hashimi, Vaishnavi Venugopalan, Maren Rehders, Naphannop Sereesongsaeng, Zeynep Hein, Sebastian Springer, Ekkehard Weber, Dagmar Führer, Matthew S. Bogyo, Christopher J. Scott, Roberta E. Burden, Klaudia Brix

**Affiliations:** 1 Department of Life Sciences and Chemistry, Jacobs University Bremen, D-28759 Bremen, Germany; a.alhashimi@jacobs-university.de (A.A.-H.); v.venugopalan@jacobs-university.de (V.V.); m.rehders@jacobs-university.de (M.R.); z.hein@jacobs-university.de (Z.H.); s.springer@jacobs-university.de (S.S.); 2 School of Pharmacy, Queen’s University Belfast, Belfast BT9 7BL, UK; nsereesongsaeng01@qub.ac.uk (N.S.); r.burden@qub.ac.uk (R.E.B.); 3 Institute of Physiological Chemistry, Martin Luther University Halle-Wittenberg, D-06114 Halle-Saale, Germany; weberekkehard123@gmail.com; 4 Klinik für Endokrinologie, Diabetologie und Stoffwechsel, Universitätsklinikum Essen (AöR), Universität Duisburg-Essen, D-45177 Essen, Germany; dagmar.fuehrer@uk-essen.de; 5 Department of Pathology, Stanford University School of Medicine, Stanford, CA 94305-5324, USA; mbogyo@stanford.edu; 6 Patrick G. Johnston Centre for Cancer Research, School of Medicine, Dentistry and Biomedical Sciences, Queen’s University Belfast, Belfast BT9 7BL, UK; c.scott@qub.ac.uk

**Keywords:** cysteine cathepsins, green fluorescent protein tagging, protein trafficking, secretion, thyroid epithelial cells, thyroid stimulating hormone

## Abstract

The significance of cysteine cathepsins for the liberation of thyroid hormones from the precursor thyroglobulin was previously shown by in vivo and in vitro studies. Cathepsin L is most important for thyroglobulin processing in mice. The present study aims at specifying the possible contribution of its closest relative, cysteine cathepsin L2/V, to thyroid function. Immunofluorescence analysis on normal human thyroid tissue revealed its predominant localization at the apical plasma membrane of thyrocytes and within the follicle lumen, indicating the secretion of cathepsin V and extracellular tasks rather than its acting within endo-lysosomes. To explore the trafficking pathways of cathepsin V in more detail, a chimeric protein consisting of human cathepsin V tagged with green fluorescent protein (GFP) was stably expressed in the Nthy-ori 3-1 thyroid epithelial cell line. Colocalization studies with compartment-specific markers and analyses of post-translational modifications revealed that the chimeric protein was sorted into the lumen of the endoplasmic reticulum and subsequently transported to the Golgi apparatus, while being N-glycosylated. Immunoblotting showed that the chimeric protein reached endo-lysosomes and it became secreted from the transduced cells. Astonishingly, thyroid stimulating hormone (TSH)-induced secretion of GFP-tagged cathepsin V occurred as the proform, suggesting that TSH upregulates its transport to the plasma membrane before it reaches endo-lysosomes for maturation. The proform of cathepsin V was found to be reactive with the activity-based probe DCG-04, suggesting that it possesses catalytic activity. We propose that TSH-stimulated secretion of procathepsin V is the default pathway in the thyroid to enable its contribution to thyroglobulin processing by extracellular means.

## 1. Introduction

Cathepsin V is a member of the human cysteine cathepsins comprising cathepsins B, C, F, H, K, L, O, S, L2/V, W and X/Z [1,2]. Cathepsin V is also referred to as cathepsin L2 due to its extensive primary sequence identity of 80% with ubiquitously expressed cathepsin L [1]. Human cathepsin L and V encoding genes are located on chromosome 9 at locus q21-q22, indicating that both enzymes arose by gene duplication from an ancestral cathepsin L-like precursor [1]. Cathepsin V was identified as the *Stratum corneum* thiol protease (SCTP) that is secreted from terminally differentiated corneocytes and able to degrade desmocollin extracellularly at desmosomes, indicating its role in desquamation [3]. Cathepsin V was also found to be expressed in corneal epithelium and in the testis as well as in the thymus, where it is believed to be involved in endosomal invariant chain processing during antigen presentation [4,5,6].

Pathologically, cathepsin V overexpression correlates with hyperproliferation in various human cancers including breast, colorectal, hepatocellular, ovarian and renal cell carcinomas [5,7]. The function of cathepsin V in triggering hyperproliferation is possibly explained by specific forms reaching the nucleus of carcinoma cells [8]. More specifically, we found that an N-terminally truncated specific form of cathepsin V was often sorted to the nuclei of cells in cold nodules and it was present in the nuclei of follicular and papillary thyroid carcinoma cells, while only occasionally being detected within nuclei of thyrocytes in non-cancerous tissue, namely, hot nodules and goiter [8]. Thus, N-terminally truncated cathepsin V is sorted to the nuclear compartment of thyroid carcinoma cells in particular, and it promotes cell proliferation [8]. In clear contrast, full-length cathepsin V was not sorted to the nucleus but was detected in the compartments of the secretory pathway of thyroid epithelial and carcinoma cells consistent with the existence of an N-terminal signal peptide [8]. However, the role of full-length cathepsin V in thyroid physiology has not been studied in sufficient detail, and its trafficking pathways upon TSH stimulation of thyrocytes remained elusive as of yet.

It is well known that the full-length forms of cysteine cathepsins B, K, L and S are important to maintain proper thyroid function, because they are involved in proteolytic processing and degradation of thyroglobulin (Tg) for thyroid hormone (TH) liberation [9,10,11,12,13]. Tg is stored within the thyroid follicle lumen in covalently cross-linked form as so-called Tg-globules reaching diameters of up to 120 μm, which considerably surpass the dimensions of single thyrocytes [14]. Therefore, luminal Tg cannot be internalized as an entity by thyrocytes, implying the need for extracellular solubilization prior to endocytosis and complete degradation of Tg within endo-lysosomes [13]. In the human thyroid, extra- and intracellular proteolytic cleavage of Tg is likely facilitated by the cysteine cathepsins B, K, L, and S acting even in the neutral and oxidizing conditions of the follicle lumen [10]. It is not known as of yet whether cathepsin V contributes to Tg processing and how it is localized in thyroid tissue. Therefore, this study seeks to understand the significance of cathepsin V in human thyroid physiology using studies on thyroid epithelial cells in situ and in vitro. The distribution of cathepsin V in thyroid tissue was investigated by immunofluorescence microscopy, revealing its noticeable extra- and pericellular localization in situ. These findings suggested functions of cathepsin V in Tg-processing by extracellular means, and highlighted the importance of studying its trafficking pathways in more detail. Thus, using thyrocytes that express enhanced green fluorescent protein (eGFP)-tagged cathepsin V (hCV-eGFP), intracellular transport routes and post-translational modifications of the hCV-eGFP chimeric protein as well as its secretion into the extracellular space were investigated in stably transduced Nthy-ori 3-1 cells (Nthyori-CV), which we established recently [8]. In addition, the effect of TSH on the expression and secretion of hCV-eGFP was investigated in vitro using this unique cell model. Nthy-ori 3-1 cells serve as an appropriate representative of thyrocytes, because this cell line has been derived from normal thyroid tissue and it responds to TSH by iodine accumulation, which is a unique feature of thyroid epithelial cells and verifies the presence of a functional TSH receptor as well as proper G protein signaling [15,16,17]. Indeed, procathepsin V becomes secreted upon TSH stimulation of Nthyori-CV cells by following the secretory pathway to the plasma membrane. This trafficking of hCV-eGFP upon TSH stimulation is in stark contrast to cysteine cathepsin trafficking observed in rodent thyrocytes, where TSH acts upon endo-lysosomes. The TSH-stimulated procathepsin V secretion from human thyrocytes is therefore novel and has not been described before, highlighting the complexity of the cellular and molecular pathways enabling Tg processing for TH liberation in the thyroid gland.

## 2. Results

### 2.1. Localization of Cathepsin V in Human Thyroid Tissue

**Figure 1:**
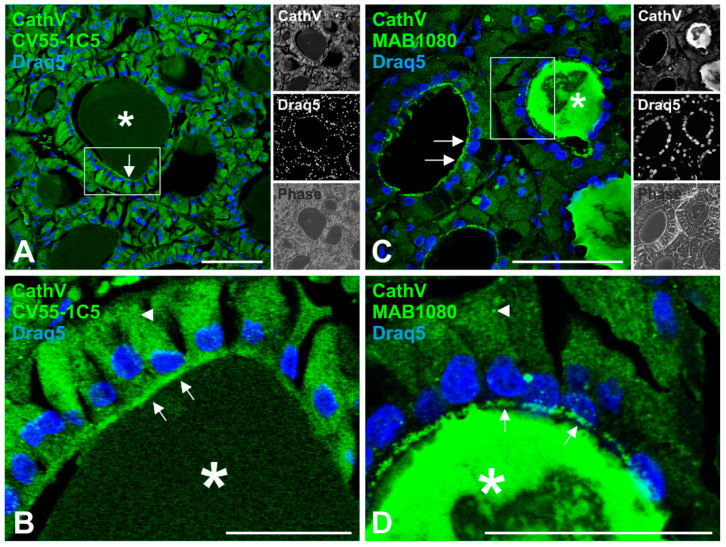
Immunohistochemistry of cysteine cathepsin V in human thyroid tissue. Human thyroid tissue sections were immunolabeled with different cathepsin V-specific antibodies (green signals), namely, anti-human cathepsin V CV55 1C5 (**A**,**B**) that exclusively immunoreacts with the proform of cathepsin V, and anti-human cathepsin V MAB1080 (**C**,**D**) recognizing both the pro- and mature cathepsin V forms. Nuclei were counter-stained with Draq5™ (blue signals). Single-channel fluorescence and corresponding phase contrast micrographs are depicted as indicated. Cathepsin V-immunopositive signals were detected within the cytoplasm (arrowheads), at the apical plasma membrane (arrows), and dispersed within the follicle lumen (asterisks). Rectangular boxes in A and C denote regions magnified in B and D, respectively. Scale bars represent 50 μm.

To study the distribution of cathepsin V in human thyroid follicles, tissue sections were immunostained with different cathepsin V-specific antibodies. Namely, the staining pattern of anti-human procathepsin V CV55-1C5, that exclusively recognizes the proform, was compared with that of anti-human cathepsin V MAB1080, which immunoreacts with both the pro- and mature forms. The thyroid tissue exhibited normal histomorphology with intact follicle structure, i.e., follicles are composed of a monolayer of polarized thyrocytes surrounding a lumen in which Tg is stored (Figure 1 asterisks), thus representing normal thyroid tissue in physiological state. Immunostaining with anti-procathepsin V CV55-1C5 antibodies revealed reticular cytoplasmic staining (Figure 1A,B arrowhead) in addition to staining at the apical plasma membrane (Figure 1A,B arrows) and moderate immunoreactions over the follicle lumen (Figure 1A,B asterisks). Intense immunofluorescence signals at the apical plasma membrane (Figure 1C,D arrows), intra-luminal staining (Figure 1C,D asterisks), and punctuate cytoplasmic staining was observed in sections immunolabeled with anti-cathepsin V MAB1080 antibodies (Figure 1C,D). The presence of cathepsin V within the thyroid follicle lumen is in accordance with our previous findings that reported the secretion of cysteine cathepsins into the lumen of human thyroid follicles [10]. Vesicular staining resembling endo-lysosomes was detected in thyroid tissue sections upon labeling with both cathepsin V antibodies, but it was not as prominent as might be expected for an endo-lysosomal enzyme (Figure 1). As expected for normal thyroid tissue, there were almost no nuclei stained with either antibody. The results provoked the questions (i) whether cathepsin V is sorted to endo-lysosomal compartments of thyroid epithelial cells at all, and (ii) which form of cathepsin V is secreted and present in the thyroid follicle lumen.

### 2.2. Cathepsin V Is Associated with the Plasma Membrane of Thyroid Epithelial Cells

**Figure 2:**
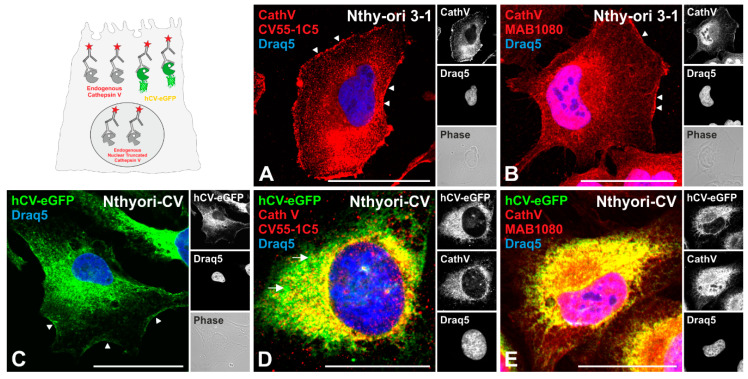
Subcellular localization of endogenous cathepsin V and eGFP-tagged full-length cathepsin V chimeric protein in thyroid epithelial cells. Non-transduced Nthy-ori 3-1 (**A**,**B**) and transduced Nthyori-CV cells (**C**–**E**) were immunostained with different cathepsin V antibodies (red signals), i.e., anti-human cathepsin V CV55-1C5 that exclusively immunodetects the proform of cathepsin V (**A**,**D**) and anti-human cathepsin V MAB1080 recognizing both, the pro- and mature cathepsin V forms (**B**,**E**). Green channels (**C**–**E**) represent fluorescence signals of hCV-eGFP. Yellow signals are indicative of colocalizing signals from cathepsin V antibodies and hCV-eGFP. The sketch (upper left panel) outlines this approach schematically. Nuclei were counter-stained with Draq5™ (blue signals). Pink signals are indicative of colocalizing signals from cathepsin V antibodies and nuclear Draq5™ staining. Single-channel fluorescence and corresponding phase contrast micrographs are depicted as indicated. Arrowheads denote immunoreactive and hCV-eGFP-derived signals at the cell surface, while arrows point to vesicular hCV-eGFP signals in Nthyori-CV cells. Scale bars represent 50 μm.

To inspect more closely how cathepsin V is transported within thyrocytes, human Nthy-ori 3-1 thyroid epithelial cells were immunolabeled with anti-procathepsin V CV55-1C5 and anti-cathepsin V MAB1080 antibodies. Immunostaining with both antibodies revealed a reticular staining pattern of cathepsin V within the cytoplasm (Figure 2A,B) in addition to staining of the cell surface (Figure 2A,B arrowheads). However, no obvious cathepsin V-immunopositive signals were observed within vesicles, indicating scarcity of this cysteine cathepsin in endo-lysosomal compartments of Nthy-ori 3-1 cells. In addition, cathepsin V MAB1080 antibodies stained an N-terminally truncated specific form of cathepsin V in the nuclei of Nthy-ori 3-1 cells (Figure 2B) as expected and described by us previously [8].

In the current study, we further focused on the non-nuclear form of cathepsin V, that is, full-length cathepsin V, which would most possibly follow the canonical trafficking pathway of endo-lysosomal enzymes from the rough endoplasmic reticulum (rER) lumen through the Golgi apparatus to endo-lysosomes due to its targeting and sorting sequences. Analysis of the transport routes of full-length cathepsin V in Nthy-ori 3-1 cells by using antibodies has limitations however, because MAB1080 antibodies cannot discriminate between the non-nuclear and nuclear forms of endogenous cathepsin V, i.e., it recognizes both the full-length and N-terminally truncated forms, while CV55-1C5 antibodies do not immuno-react with the mature cathepsin V forms [8]. Therefore, we sought to generate a cell line expressing full-length cathepsin V, which is C-terminally tagged with GFP, and otherwise bears all targeting and sorting sequences of the wild type full-length cathepsin V. Therefore, Nthy-ori 3-1 cells were transduced to stably express human full-length cathepsin V fused with eGFP (hCV-eGFP) as previously described [8], henceforth referred to as Nthyori-CV cells.

The chimeric protein hCV-eGFP revealed the same localization as the endogenous, non-nuclear cathepsin V when Nthyori-CV cells were inspected by fluorescence microscopy before and after staining with CV55-1C5 and MAB1080 antibodies (Figure 2C cf. Figure 2D,E respectively). Thus, hCV-eGFP became predominantly detectable in reticular structures, and it was found to a lesser extent in vesicles distributed throughout the cytoplasm of Nthyori-CV cells (Figure 2C). Importantly, Nthyori-CV cells exhibited cell surface localization of eGFP-tagged full-length cathepsin V (Figure 2C arrowheads). Immunolabeling of Nthyori-CV cells with CV55-1C5 antibodies revealed that the fluorescence signals of the chimeric protein hCV-eGFP and the anti-procathepsin V derived immunofluorescence signals were colocalized in structures of the perinuclear region (Figure 2D yellow signals). However, few eGFP-positive vesicles, which were scattered throughout the cytoplasm, were not immunostained with CV55-1C5 antibodies (Figure 2D arrows), suggesting that these vesicles possibly contained a GFP-tagged mature form of cathepsin V that could not be recognized by anti-procathepsin V antibodies. In contrast, in the reticular structures in the cytoplasm of Nthyori-CV cells, maximum co-localization was observed between anti-cathepsin V MAB1080 antibody-stained structures and those containing hCV-eGFP chimeric protein (Figure 2E yellow signals). The results underlined that Nthyori-CV cells are a suitable in vitro model to study the sorting and trafficking of full-length cathepsin V in thyroid epithelial cells.

### 2.3. The Chimeric Protein hCV-eGFP Undergoes N-Linked Glycosylation in Thyroid Epithelial Cells

**Figure 3:**
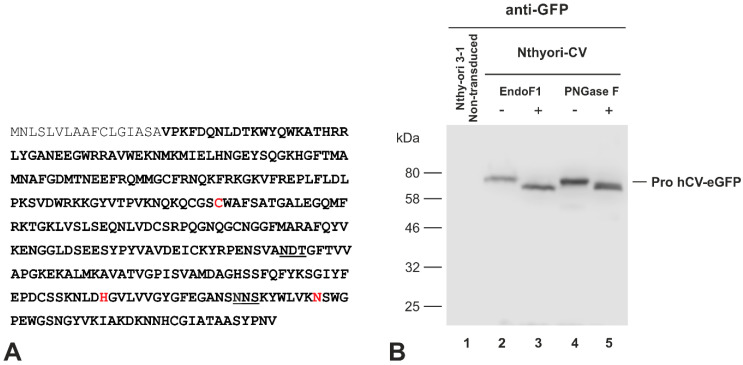
N-glycosylation state of hCV-eGFP chimeric protein in thyroid epithelial cell lines. (**A**) The amino acid sequence of human cathepsin V (UniProt accession number O60911) contains two potential N-glycosylation sites of the motif “N-X-S/T-NOT P” at positions Asn-221 and Asn-292 (underlined). The active site residues Cys-138, His-277 and Asn-301 indicative of cysteine peptidases are highlighted in red. The propeptide is indicated in gray font. (**B**) Immunoblots of lysates prepared from Nthy-ori 3-1 control (lane 1) or Nthyori-CV cells, treated without (lanes 2 and 4) or with (lanes 3 and 5) EndoF1 or PNGase F, were probed with anti-GFP antibodies. Molecular mass markers are indicated in the left margin. Faster migration due to glycosidase-mediated reduction in the molecular mass of the proform of hCV-eGFP chimeras was indicative of the removal of N-linked glycans.

According to its amino acid sequence, the cysteine cathepsin V harbors two putative N-glycosylation sites at residues Asn-221 and Asn-292, respectively (Figure 3A underlined). Because N-glycosylation is important for protein stability and trafficking, we explored whether the eGFP-tagged full-length cathepsin V chimeric protein might become N-glycosylated in the transduced thyroid epithelial cells. Therefore, lysates of Nthyori-CV cells were incubated with glycosidases, namely, PNGase F or EndoF1, before immunoblotting. Staining with GFP-specific antibodies revealed that the bands at approx. 64 kDa, representing the proform of the hCV-eGFP chimeric protein (Figure 3B, lanes 2 and 4, respectively), migrated faster after treatment with both EndoF1 and PNGase F (Figure 3B, lanes 3 and 5, respectively). The results indicated that hCV-eGFP chimeric protein undergoes N-glycosylation in the transduced Nthyori-CV cells. It is worth noting that both glycosidases were productive in de-glycosylation of hCV-eGFP, which led us to conclude that the chimeric protein remained in an EndoF-sensitive form in the Nthyori-CV cells. Since EndoF1 recognizes asparagine-linked or free high mannose and hybrid carbohydrate modifications, but not complex oligosaccharides, the data suggested that hCV-eGFP chimeric protein contains oligo mannosyl- or hybrid-type glycans. We further reasoned that EndoF sensitivity of hCV-eGFP was either due to its trapping in the ER and failure to reach the Golgi apparatus, or alternatively, due to the failure of N-glycan maturation in Nthyori-CV cells despite regular trafficking along the secretory pathway. Therefore, we next investigated the intracellular trafficking route of hCV-eGFP in more detail by performing co-localization experiments with compartment-specific markers.

### 2.4. The Transport Route of the Chimeric Protein hCV-eGFP in Thyroid Epithelial Cells

**Figure 4:**
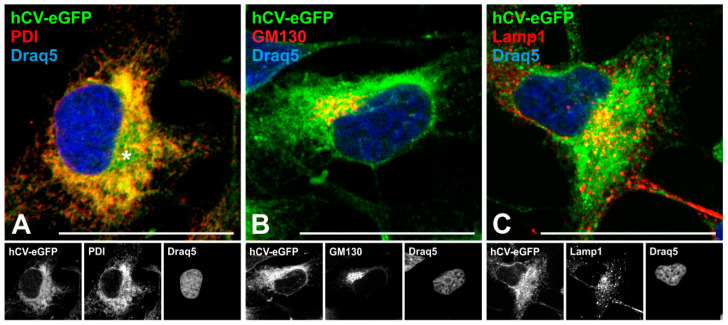
Trafficking of eGFP-tagged full-length cathepsin V in thyroid epithelial cells. Confocal laser scanning micrographs of Nthyori-CV cells expressing hCV-eGFP chimeras ((**A**–**C**), green signals) after immunolabeling with antibodies against PDI (**A**), GM130 (**B**) and Lamp1 (**C**) proteins residing in the ER, at the cytosolic face of Golgi cisternae and vesicles, and in endo-lysosomes, respectively (red signals). Yellow signals are indicative of co-localization. Nuclei were counter-stained with Draq5™ (blue signals). Single-channel fluorescence micrographs are depicted in the bottom panels as indicated. Scale bars represent 50 μm.

To analyze trafficking and sorting of eGFP-tagged full-length cathepsin V in more detail, Nthyori-CV cells were immunostained with compartment-specific markers, namely, by using antibodies against the ER-resident protein disulfide isomerase (PDI), the cis-Golgi matrix protein GM130, and the lysosome-associated membrane protein 1 (Lamp1). The results revealed co-localization of hCV-eGFP chimeric protein with PDI in ER-like reticular distribution patterns (Figure 4A, yellow signals). In addition, eGFP-tagged full-length cathepsin V was observed in PDI-negative cisterna-like structures in the peri-nuclear region (Figure 4A, asterisk, green signals) which were identified as belonging to the Golgi apparatus by the pronounced co-localization with GM130 (Figure 4B, yellow signals). The chimeric hCV-eGFP protein was also found to co-localize with Lamp1-positive vesicles in Nthyori-CV cells but only in some endo-lysosomes (Figure 4C, yellow signals). The results demonstrated that the hCV-eGFP chimeric protein is synthesized at the ER and targeted for entry into its lumen before being transported to the Golgi apparatus and delivery to its final destination, the endo-lysosomal compartments. Co-localization of hCV-eGFP with markers of early secretory pathway compartments was notably more extensive than with endo-lysosomal markers.

**Figure 5:**
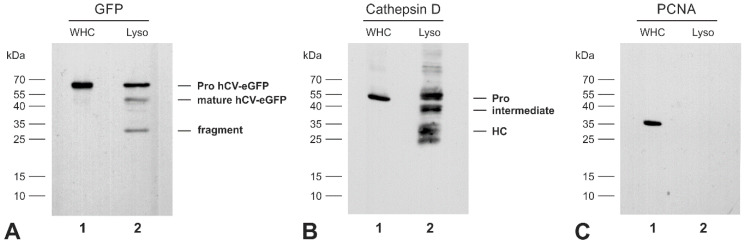
Presence of mature hCV-eGFP chimeric protein within endo-lysosomal fractions. Immunoblots of lysates prepared from whole cells (WHC, lanes 1, respectively) or endo-lysosome enriched fractions (Lyso, lanes 2, respectively) of Nthyori-CV cells were probed with anti-GFP (**A**), anti-cathepsin D (**B**), and anti-PCNA antibodies (**C**), respectively. Molecular mass markers are indicated in the left margins (**A**–**C**). Only the proforms (pro) of hCV-eGFP and cathepsin D were detected in the whole cell lysates of Nthyori-CV cells ((**A**,**B**), lanes 1, respectively). In addition to the proform, the endo-lysosomal fractions of Nthyori-CV cells contained the processed mature form of hCV-eGFP and its degradation fragment ((**A**), lane 2). The molecular forms of cathepsin D, i.e., proform (Pro), intermediate, and heavy chain of two-chain cathepsin D (HC), were detected in the endo-lysosomal fractions ((**B**), lane 2). Immunoblotting with anti-PCNA antibodies confirmed the purity of the endo-lysosomal fractions (**C**).

Since the immunofluorescence studies revealed only partial co-localization of hCV-eGFP chimeras with Lamp1, we sought to verify biochemically whether it reaches endo-lysosomes. Hence, Nthyori-CV cells were homogenized and endo-lysosome-enriched fractions were isolated by differential centrifugation. Lysates of whole cells and endo-lysosomal fractions prepared from Nthyori-CV cells were blotted with GFP-specific antibodies, resulting in immunorecognized bands of approximately 64 kDa, corresponding to the predicted molecular mass of the proform of hCV-eGFP (Figure 5A, lanes 1 and 2). However, additional bands at approximately 51 kDa and 30 kDa, corresponding to the expected molecular masses of the mature form of hCV-eGFP and its degradation fragment, respectively, were detected in the endo-lysosomal fractions of Nthyori-CV cells (Figure 5A, lane 2). Immunoblotting for lysosomal cathepsin D and proliferating cell nuclear antigen (PCNA) confirmed the purity of the endo-lysosomal fractions (Figure 5B,C). A band representing procathepsin D was detected in the whole cell lysate (Figure 5B, lane 1), while different molecular forms of cathepsin D, i.e., proform, intermediate, and heavy chain of two-chain cathepsin D, were abundant in the endo-lysosomal fractions of Nthyori-CV cells (Figure 5B, lane 2). PCNA protein was present in the whole cell lysate but absent from endo-lysosomal fractions of Nthyori-CV cells (Figure 5C, lane 1) as expected. The results demonstrated the presence and enrichment of hCV-eGFP chimeras as pro- and mature forms in endo-lysosomal fractions of Nthyori-CV cells, indicating the transport of hCV-eGFP to the predicted final destination for maturation in endo-lysosomal compartments.

### 2.5. The Chimeric Protein hCV-eGFP Is Secreted from Thyroid Epithelial Cells

**Figure 6:**
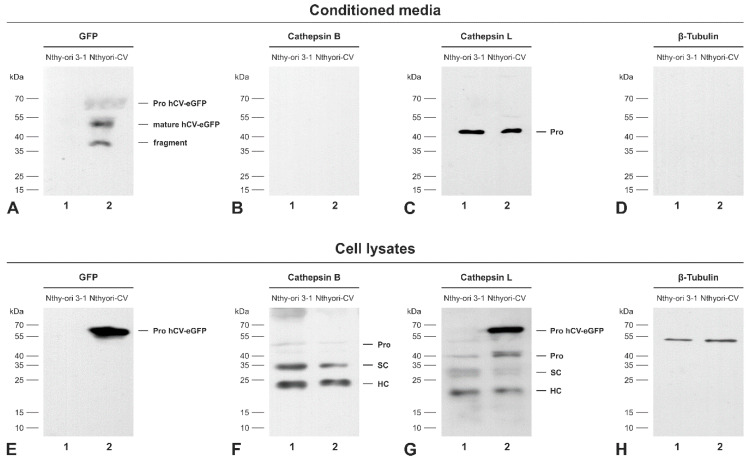
Secretion of hCV-eGFP chimeric protein from thyroid epithelial cells. TCA precipitated proteins of 24 h conditioned media (**A**–**D**) collected from confluent Nthy-ori 3-1 (lanes 1) or Nthyori-CV cell cultures (lanes 2) and the corresponding cell lysates (**E**–**H**) were immunoblotted with anti-GFP (**A**,**E**), anti-cathepsin B (**B**,**F**), anti-cathepsin L (**C**,**G**), and anti-β-tubulin (**D**,**H**) antibodies. The conditioned media of Nthyori-CV cells contained mainly the processed mature form of hCV-eGFP in addition to its proform and a degradation fragment of approximately 37 kDa ((**A**), lane 2). Cathepsin L was found extracellularly only as proform ((**C**), lanes 1 and 2). hCV-eGFP was present predominantly in its proform in the lysate of Nthyori-CV cells ((**E**), lane 2). However, the expected molecular forms of cathepsins B and L, namely the proform (pro), single-chain (SC), and the heavy chain (HC) of the two-chain forms were detected in the lysates of both Nthy-ori 3-1 and Nthyori-CV cells ((**F**,**G**), respectively). Molecular mass markers are indicated in the left margins. No anti-GFP bands were observed in conditioned media or lysates of non-transduced Nthy-ori 3-1 cells ((**A**,**E**), lane 1), confirming specificity of the GFP-specific antibodies. Immunoblots with anti-β-tubulin antibodies (**D**,**H**) confirmed that conditioned media were not contaminated by cell debris, and that the TCA precipitates represented only the proteins secreted from the investigated cells.

In order to address the question as to whether cathepsin V is secreted into the extracellular space of the transduced thyrocytes in steady state, TCA-precipitated proteins of conditioned media of confluent Nthyori-CV or non-transduced Nthy-ori 3-1 control cells (Figure 6) were analyzed by immunoblotting. A single band at 64 kDa, representing the proform of hCV-eGFP, was prominent in the cell lysates of Nthyori-CV cells but not observed in lysates of non-transduced cells (Figure 6E), whereas this band was also present in low amounts in the conditioned media of Nthyori-CV cells (Figure 6A, lane 2). Anti-GFP positive bands with apparent molecular masses of 51 and 37 kDa, corresponding to the mature form and to an hCV-eGFP-derived fragment, respectively, were additionally detected in the conditioned media of Nthyori-CV cells with higher intensity (Figure 6A, lane 2).

Recently, we showed that Fisher rat thyroid epithelial cells secrete procathepsin L to much higher extents than procathepsin B [18], which was confirmed for Nthy-ori 3-1 cells in this study (Figure 6B,C,F,G, lanes 1, respectively), indicating that cysteine cathepsin trafficking is similar in human and rat thyroid epithelial cells. Transduced Nthyori-CV cells exhibited a similar pattern of cathepsin B and L secretion as non-transduced Nthy-ori 3-1 control cells (Figure 6B,C,F,G, lanes 2, respectively), while the proform of hCV-eGFP chimeras was also recognized by anti-cathepsin L antibodies in the lysates of transduced cells (Figure 6G, lane 2), indicating the similarity of cathepsins L and V.

We concluded that thyroid epithelial cells can secrete the proform of eGFP-tagged cathepsin V, in principle. However, the predominant occurrence of mature hCV-eGFP in the conditioned media of Nthyori-CV cells could be explained either by extracellular processing of secreted procathepsin V-eGFP to the mature form, or alternatively, by the secretion of mature chimeric protein recruited out of endo-lysosomes.

### 2.6. Secretion of the Proform of hCV-eGFP Is Triggered by TSH Stimulation

It is well known that proteolytic Tg processing for TH liberation by extra- and intracellular means is regulated by TSH [13]. In addition, we have previously reported that TSH stimulates the retrieval of mature cathepsin B out of endo-lysosomes for subsequent secretion from the FRTL-5 rat thyroid epithelial cell line [19,20]. Hence, we were interested in investigating whether TSH alters the rates of hCV-eGFP chimeric protein secretion from Nthyori-CV cells. Therefore, confluent Nthyori-CV cells were stimulated with 100 µU/mL TSH for up to 24 h, and conditioned media were analyzed by immunoblotting, in comparison with those of non-stimulated Nthyori-CV cells, to have a measure of steady state 24-h-secretion extents.

**Figure 7:**
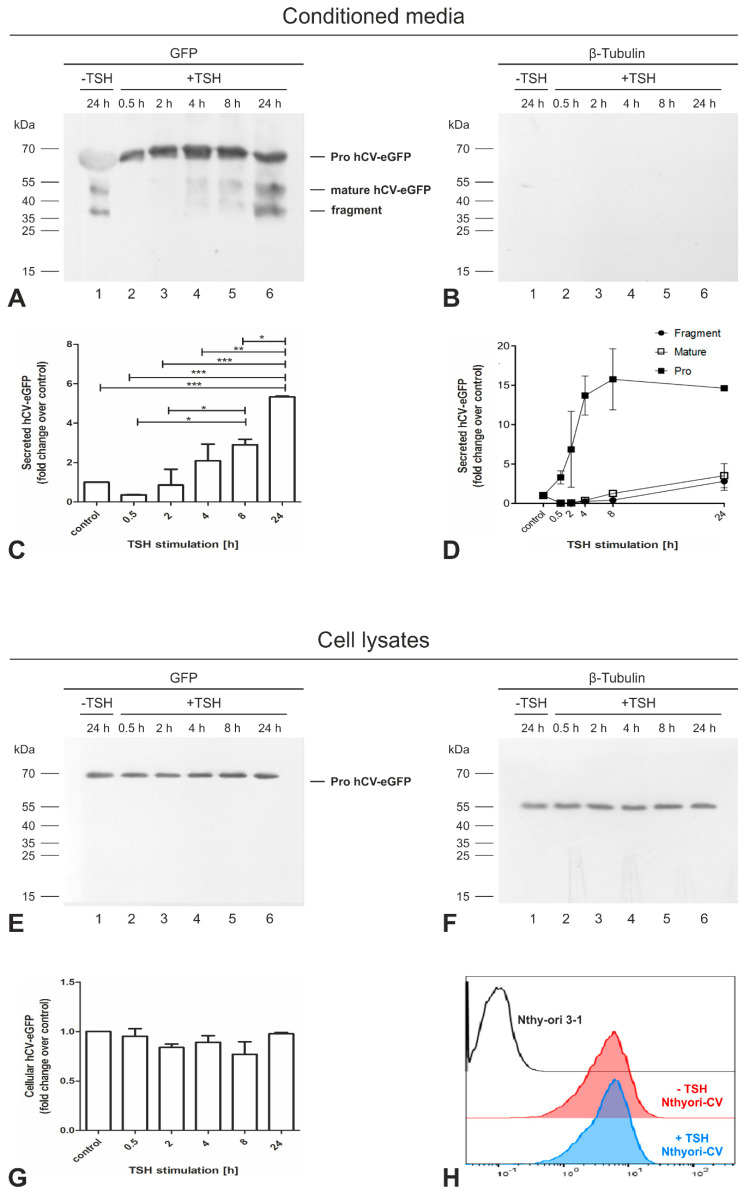
TSH regulates the secretion of the proform of hCV-eGFP chimeric protein from thyroid epithelial cells. Proteins precipitated with TCA from the conditioned media of confluent Nthyori-CV cells stimulated with 100 µU/mL TSH for different time intervals, as indicated ((**A**,**B**), lanes 2–6), and corresponding cell lysates ((**E**,**F**), lanes 2–6) were separated by SDS-PAGE. The 24 h conditioned media collected from confluent Nthyori-CV cells and its respective cell lysate represented the non-stimulated control (lanes 1). After blotting, proteins were immunolabeled with anti-GFP (**A**,**E**). To verify that TCA-precipitated proteins were not contaminated with cellular debris, the same membrane was stripped and reblotted with anti-β-tubulin antibodies (**B**,**F**). No bands corresponding to β-tubulin were seen in any of the conditioned media precipitates, verifying that the secreted proteins of Nthyori CV cells exclusively were analyzed. Molecular mass markers are indicated in the left margins. The total amounts (**C**) and the amounts of proform and processed forms, i.e., the mature form and the derived fragment (**D**), of secreted hCV-eGFP chimeric protein were quantified by densitometry. The amounts of intracellular hCV-eGFP chimeric protein were also quantified and normalized to β-tubulin (**G**) and remained unchanged upon TSH treatment indicating unaffected de novo-biosynthesis rates. The intracellular amounts of hCV-eGFP in non-stimulated (red) and 24 h TSH stimulated (blue) Nthyori-CV cells were determined using flow cytometry (**H**), also revealing comparable expression in non- and TSH-stimulated cells. Fold changes were calculated in comparison with non-stimulated controls (**C**,**D**,**G**). Data are depicted as means + SD in (**C**,**G**), and as means ± SD for the processed forms in (**D**). Levels of significance were determined by one-way ANOVA, followed by Tukey post hoc tests, and are indicated as * for *p* < 0.05, ** for *p* < 0.01, and *** for *p* < 0.001, respectively. The experiments were repeated twice.

Immunoblotting with anti-GFP antibodies revealed the presence of a distinct band at approximately 64 kDa representing the proform of hCV-eGFP in the conditioned media of TSH-stimulated cell cultures at all time intervals (Figure 7A, lanes 2–6), while only a faint band at 64 kDa was observed in the conditioned medium of non-stimulated Nthyori-CV cells (Figure 7A, lane 1; cf. Figure 6). However, the processed form of eGFP-tagged full-length cathepsin V protein, represented by the band at 51 kDa, corresponding to the mature form, and an hCV-eGFP-derived fragment of 37 kDa were indeed detected in the 24 h conditioned media of TSH-stimulated as in non-stimulated cells (Figure 7A, lanes 1 and 6). Densitometric analysis of the anti-GFP positive bands showed that the amounts of TCA-precipitable chimeric protein increased in the conditioned media of TSH-stimulated cells over the time course of 24 h, and reached higher levels than in non-stimulated cell cultures (Figure 7C). Moreover, secretion of the proform of hCV-eGFP was triggered already within 0.5 h of TSH stimulation and gradually increased over time, peaking at 8 h (Figure 7D), while processed forms of the chimeric protein were observed from 4 h of TSH-stimulation (Figure 7D). These results imply that Nthyori-CV cells secrete the proform of the hCV-eGFP chimeric protein in a TSH-regulated fashion into the extracellular space, where it is processed to its mature form, while also a 37-kDa-fragment is generated.

To investigate whether TSH-stimulation affects intracellular protein concentrations and/or affected the maturation of eGFP-tagged cathepsin V-chimeras, the respective lysates of TSH-stimulated and non-stimulated Nthyori-CV cells were analyzed (Figure 7E), demonstrating that only the band corresponding to the proform of hCV-eGFP was present intracellularly. The amounts of intracellular hCV-eGFP were quantified by densitometry and normalized to β-tubulin, revealing that the protein amounts of intracellular proforms of hCV-eGFP remained unaltered upon TSH stimulation for up to 24 h (Figure 7G), indicating that TSH did not largely affect the de novo biosynthesis rates of hCV-eGFP. To further confirm that long-term TSH-stimulation did not result in up-regulation of intracellular levels of hCV-eGFP, its expression in Nthyori-CV cells stimulated with TSH for 24 h was assessed using flow cytometry and compared to non-stimulated controls, revealing comparable intracellular amounts of eGFP-tagged cathepsin V protein (Figure 7H).

### 2.7. The Activity-Based Probe DCG-04 Recognizes the Proform of eGFP-Tagged Cathepsin V Chimeric Protein

**Figure 8:**
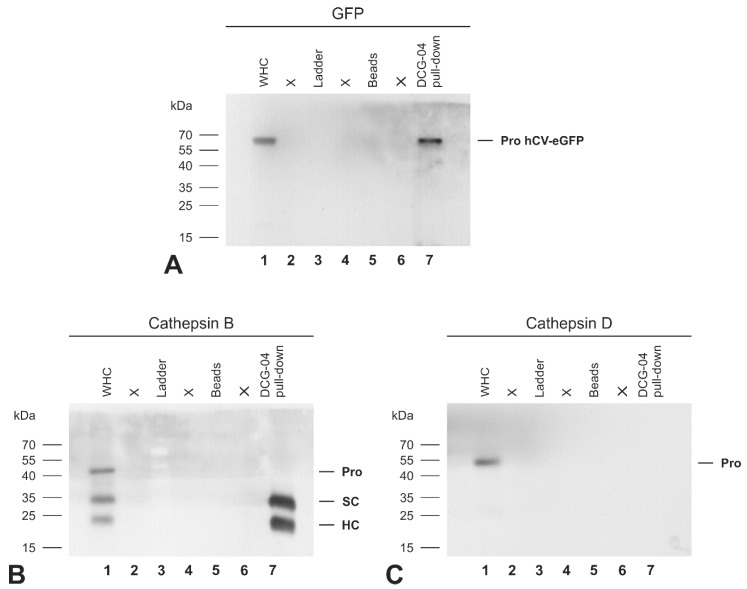
Detecting the proform of hCV-eGFP chimeric protein with the activity-based probe DCG-04. Lysates (WHC, lanes 1, respectively) were prepared from Nthyori-CV cells in the presence of biotinylated DCG-04, which requires an accessible active site in order to bind covalently to proteolytically active cysteine peptidase forms in a 1:1 ratio. The whole cell lysate was then incubated with streptavidin beads to pull down DCG-04-labeled proteins (DCG-04 pull down, lanes 7, respectively), representing active cysteine peptidase forms. Immunoblots were probed with anti-GFP (**A**), anti-cathepsin B (**B**), and anti-cathepsin D (**C**) antibodies. Molecular mass markers are indicated in the left margins. It was found that the active site of the proform of the hCV-eGFP chimeric protein was fully accessible for binding to DCG-04, indicating its proteolytic activity ((**A**), lane 7). The whole cell lysate contained the expected molecular forms of cathepsin B, i.e., proform (pro), single chain (SC), and the heavy chain (HC) of the two-chain form ((**B**), lane 1), while only the mature forms of cathepsin B were detected in the DCG-04 pull down ((**B**), lane 7), verifying its exclusive binding to the active site. Procathepsin D was detected in the whole cell lysate ((**C**), lane 1), but not in the DCG-04 pull down ((**C**), lane 7), verifying the specificity of the activity-based probe DCG-04 for cysteine peptidases.

Cysteine cathepsins are synthesized as zymogens and are processed to their mature forms by the removal of their pro-peptides, which play a critical role in regulating the proteolytic activities, e.g., by blocking access to the active site [21,22,23,24]. Thus, proteolytic procathepsin maturation is typically considered essential for the enzymes to acquire full proteolytic activity. To gain insight into the activity of the different molecular forms of the hCV-eGFP chimera, accessibility to its active site was probed by using the activity-based probe DCG-04 that covalently binds to active cysteine peptidases in a 1:1 ratio, only, when the active site is accessible. Therefore, lysates of Nthyori-CV cells were prepared in the presence of biotinylated DCG-04. Taking advantage of the biotin tag present on DCG-04, proteins that became covalently bound to the DCG-04 were then pulled-down using streptavidin-coated beads and immunoblotted with anti-GFP antibodies. Whole cell lysates, not incubated with streptavidin-coated beads, were analyzed as controls. As observed before (see Figure 6E), a single band representing the proform of the hCV-eGFP chimeric protein was observed in the whole cell lysates of Nthyori-CV cells (Figure 8A, lane 1). Interestingly, the same 64-kDa band was detected in the DCG-04 pull-down (Figure 8A, lane 7), implying that it was the proform of hCV-eGFP chimeras that was accessible for the DCG-04 probe. To confirm the efficiency and specificity of the DCG-04 pull-down, i.e., solely revealing active cysteine proteases, the anti-GFP immunoblot was subsequently stripped and re-incubated with anti-cathepsin B and anti-cathepsin D antibodies. The three expected molecular forms of cysteine cathepsin B were identified in the cell lysates, namely proform (pro), single-chain (SC) and the heavy chain (HC) of the two-chain form (Figure 8B, lane 1). On the other hand, only two bands representing single- and heavy-chain forms of cathepsin B were detected in the DCG-04 pull down, indicating the accessibility of DCG-04 to the mature forms of cathepsin B, only (Figure 8B, lane 7), and thus verifying the probe’s specificity in this experimental set-up. Immunoblotting with cathepsin D antibodies showed one band at 52 kDa, corresponding to the proform of cathepsin D, exclusively in the cell lysates (Figure 8C, lane 1). Because cathepsin D is an aspartic protease, it was not detectable in the DCG-04 pull-down (Figure 8C, lane 7), confirming that the activity-based probe DCG-04 selectively binds to active cysteine peptidases only, and does not exhibit cross-class reactivity. These results showed that despite the presence of the propeptide, hCV-eGFP was labeled by the activity-based probe DCG-04, indicating the accessibility of its active site, thereby suggesting that this chimeric protein is proteolytically active in its proform.

## 3. Discussion

### 3.1. Trafficking of Cathepsins to Endo-Lysosomes

Cathepsins are proteolytic enzymes that cleave a wide range of substrates. The general scheme of cathepsin trafficking and maturation has however been challenged, because specific forms may acquire a proteolytically active conformation in unexpected locations such as the cytosol, mitochondria, and the nucleus [8,25,26,27,28,29]. Moreover, cathepsins are secreted from various cell types in both physiological and pathological conditions [10,19,20,24,30]. Hence, cathepsin sorting and targeting depends upon the specific cell type looked at, the (patho)physiological state of a given tissue, and, at the cellular level, trafficking is directed by transcriptional and post-transcriptional regulation [23].

Overall, it is well established that cathepsins, like other endo-lysosomal enzymes, require an N-terminal signal peptide (prepeptide) to start their journey along the secretory pathway [22]. Thus, cathepsins are synthesized as pre-pro-proteins, whereby it is accepted that the prepeptide guides the nascent chains into the lumen of the rER, while the propeptides keep them in the zymogen state until the final destination, endo-lysosomes, is reached [23,24,31]. In this study, we expressed the chimeric protein hCV-eGFP containing a canonical 17-amino-acid signal peptide, which enables its entry into the rER as shown by colocalization with the ER-resident PDI. The expressed eGFP-tagged cathepsin V then became sorted to the Golgi apparatus and it reached endo-lysosomes as shown herein by immunofluorescence co-localization studies and by immunoblotting of endo-lysosomal fractions. Hence, hCV-eGFP follows the expected trafficking pathway, in principle.

Canonically, post-translational modifications like N-glycosylation and mannose-6 phosphorylation are critical determinants of endo-lysosomal protein trafficking [21,22,23,31,32,33,34]. Cathepsin V, in particular, contains two N-glycosylation consensus motives (Asn-X-Ser/Thr-NOT Pro) located at Asn-221 and Asn-292 [4,5]. The significance of N-glycosylation for the transport of cathepsin V was studied in human fibrosarcoma cells, where mutational elimination of one or two N-glycosylation sites led to decreased secretion rates, while also affecting the sorting of the mutant cathepsin V forms to endo-lysosomes [35]. Here, we demonstrated that cathepsin V is N-glycosylated and remains EndoF-sensitive in human thyroid epithelial cells. Typically, glycoproteins acquire EndoF-resistance in the medial Golgi, when N-glycans are processed to complex-type structures [36,37]. However, and in contrast to this general scheme, endo-lysosomal glycoproteins are also known to pass through the medial and trans Golgi, while remaining susceptible to endoglycosidases, because they are often tagged with mannose-6 phosphate (M6P) residues blocking carbohydrate processing to hybrid and complex glycans [34,38,39,40,41]. Therefore, we propose that the chimeric protein hCV-eGFP carries oligo-mannose glycans that remain EndoF-sensitive due to its mannose-6 phosphorylation. Despite bearing the M6P recognition marker, procathepsins can still skip sorting to endo-lysosomes and follow alternative transport routes. Such alternative M6P-independent transport pathways are believed to be realized either because of the reduced availability of saturated or down-regulated cation-dependent M6P receptors (CD-MPRs) in the trans-Golgi network (TGN), or because of non-canonical glycosylation. Consequently, precursor forms of such endo-lysosomal glycoproteins would become secreted to the extracellular space as zymogens by following the default secretory pathway to the plasma membrane [21,38,39,42,43].

### 3.2. Transport Routes of Cathepsins to the Extracellular Space

Sorting of cathepsins to extracellular localizations is often associated with their overexpression, which typically coincides with pathological conditions like cancer or inflammatory diseases [44,45]. However, cathepsins are also secreted from different cell types such as, e.g., keratinocytes [46], osteoclasts [47], and thyrocytes [18,19,20], thereby participating in physiological processes like cell migration for wound healing, bone remodeling, and prohormone processing [23,48,49]. Cathepsin secretion can be induced by diverse signaling pathways. For instance, TSH signaling regulates the secretion of proteases from thyrocytes at the apical pole into the extracellular follicle lumen in order to maintain thyroid function [13,14,50]. TSH is secreted from the pituitary gland upon TH demand and it is delivered with the blood to reach TSH receptors (TSHR) at the basolateral pole of thyroid epithelial cells [51,52]. The binding of TSH to its G protein-coupled receptor activates the Gαq-phospholipase Cβ (PLCβ) signaling cascade, which, in turn, causes a rise in free calcium (Ca^2+^) concentrations [53,54]. The elevation of cytosolic Ca^2+^ triggers the fusion of endo-lysosome-derived transport vesicles with the apical plasma membrane and subsequent release of their contents into the extracellular space [19,20]. Therefore, the short-term effects of TSH result in the secretion of proteases in order to initiate the solubilization of Tg within the follicle lumen, followed by its internalization for exhaustive Tg degradation and TH liberation in endo-lysosomal compartments [8]. This concept was first proposed in previous studies by us, and it demonstrated that the secretion of mature cathepsin B from porcine or FRTL-5 rat thyrocytes followed that pathway, which was upregulated within a few hours of TSH stimulation [19,20,50]. In this study, however, we have shown that the extracellular presence of the hCV-eGFP chimeric protein was detectable already within the first 30 min of TSH stimulation and its secretion increased over time. These results led us to conclude that the secretion of cysteine cathepsins from thyrocytes is regulated by TSH, however, different transport pathways can be followed upon TSH-stimulation. While cathepsin B is recruited out of endo-lysosomes and hence follows retrograde trafficking [19,20], procathepsin V-eGFP follows the secretory pathway in anterograde fashion, that is, from the ER to the Golgi onwards to the cell surface (this study).

Upon secretion, cathepsins can associate with their substrates in the plasma membrane-near pericellular region, a scenario that explains their stabilization by protection from the non-favorable conditions of the extracellular milieu and the maintenance of proteolytic activity, respectively [30,49,55,56]. In the current study, the localization of cathepsin V in the follicle lumen and at the plasma membrane was shown in situ and in vitro, thereby making the above scenarios plausible in thyroid epithelial cells too. Since cathepsin V provides positively charged patches on its molecular surface [25], we suggest that these may facilitate the interaction of cathepsin V to negatively charged, sialylated plasma membrane constituents via electrostatic interactions. Future studies will be important to investigate the molecular nature of procathepsin V binding sites at cell surfaces, including those of thyrocytes.

### 3.3. Mechanisms of Procathepsins Activation

Cathepsin V has been reported to be optimally active at pH 5.7 toward peptide substrates like Z-Phe-Arg-MCA containing a cleavage motif used by several cysteine cathepsins including cathepsin L with a more acidic pH optimum [1,57]. Nevertheless, cysteine cathepsins are known to retain some activity at neutral pH as well, particularly when stabilized by high substrate concentrations [10,58,59]. Among the cysteine cathepsins, it was so far found that cathepsin S in particular has high stability at neutral pH [30,48,60]. In addition, cathepsin V is also stable at neutral pH, unlike its closest homologous cathepsin L [1]. In the thyroid, the situation is such that cysteine cathepsins B, K, L, and S are proteolytically active on their natural substrate Tg even under the neutral and oxidizing conditions of the follicle lumen [10]. Using an in vitro assay mimicking the in situ conditions, we showed that cathepsin S is indeed the most efficient in Tg-processing for TH liberation under such highly non-favourable conditions [10]. That study had further shown that also the other cysteine cathepsins B, K, and L cleaved their substrate at neutral pH and in an oxidizing milieu [10]. We conclude that the thyroid follicle lumen is special in that it contains a very high concentration of protease substrate, namely, covalently cross-linked Tg-globules, that stabilize cysteine cathepsins reaching this extracellular space as proforms by anterograde (this study) or as mature forms by retrograde trafficking pathways [13,19,20].

In this context it is important to remember that the removal of the pro-peptides is a crucial step in generating fully active mature cathepsins. The pro-peptide chain folds onto the surface of the zymogens in an extended conformation and runs through the active site in opposite direction to the substrate, thus blocking the access of any substrate to the active site. The interactions between the pro-peptides and the remaining portions of the pro-proteins are brought about by salt bridges, hydrogen bonding, and hydrophobic interactions, thereby maintaining the folding integrity of the pro-peptides and mediating their inhibitory effect on the zymogens [61,62,63]. Such pro-peptide interactions are weakened at low pH, leading to a conformational change in the cathepsin zymogen forms, whereby their proteolytic processing for maturation is facilitated [64,65,66]. However, disturbances of the interactions between pro-peptides and the remaining portions of the pro-cathepsins can also occur at neutral pH, especially in an extracellular milieu rich in glycosaminoglycans and other negatively charged molecules [59,67]. Once the pro-peptides are dissociated from the active-site cleft, the zymogen forms become more susceptible to proteolytic cleavage, resulting in pro-cathepsin conversion to the mature forms. Glycosaminoglycans are typical constituents of the basolateral extracellular matrix of epithelial cells, thus unlikely to be found excessively in the thyroid follicle lumen which is apposed to the apical pole of thyroid epithelial cells. In the extracellular follicle lumen, however, highly glycosylated Tg is found and its carbohydrate-rich post-translational modifications might perform a function similar to that of glycosaminoglycans of the extracellular matrix. In particular, human Tg contains chondroitin sulfate [14,68,69,70], which could perform the function of disturbing pro-peptide interactions in the zymogen forms. In addition, chondroitin sulfate modifications render Tg negatively charged, which would explain why positively charged molecules like procathepsin V interact preferentially with it in the follicle lumen of the thyroid gland.

Otherwise, pro-peptides are enzymatically removed by means of legumain-mediated processing or autocatalytically in the case of endopeptidases such as the cathepsins B, H, K, L, and S [23,65,66,71,72,73]. Regarding the activation of procathepsin V, it was reported that recombinant procathepsin V can be autocatalytically activated at acidic pH in a process that is inhibited by various cysteine protease inhibitors [1], already hinting to the possibility of enzymatic activity of this cysteine cathepsin in the zymogen form. In the current study, procathepsin V was shown to be accessible to the activity-based probe DCG-04, which strongly implies that the zymogen form of cathepsin V is proteolytically active. Interestingly, procathepsin B is also accessible to DCG-04 and can act auto-catalytically on itself [72,73]. In the thyroid gland, however, procathepsin B is sorted to endo-lysosomes and it is plausible that canonical activation predominates [19,20]. In contrast, we propose for cathepsin V that it is secreted from thyrocytes in the proform, thereby reaching the extracellular follicle lumen, where it is stabilized by the high concentration of its substrate Tg. Subsequently, procathepsin V becomes matured most likely via autocatalytic cleavage, promoted by the glycosylation modifications on Tg, and, upon TSH stimulation, via activation by other cysteine cathepsins which are then delivered to the follicle lumen in proteolytically active forms like the cathepsins B, K, or S.

### 3.4. Perspectives

Taken together, the herein described localization of procathepsin V in the thyroid follicle lumen and its secretion from thyrocytes in a TSH-stimulated fashion support the notion that cathepsin V is another cysteine cathepsin being involved in thyroglobulin processing in addition to cathepsins B, K, L, and S. Future studies are underway to determine whether thyroglobulin is a natural substrate of procathepsin V, and where precisely it is cleaved, and whether this cleavage is productively contributing to extracellular TH liberation in the thyroid follicle lumen in order to further specify the putative contribution of procathepsin V in maintaining thyroid homeostasis. Cathepsin V is, however, special among the thyroidal cysteine cathepsins in that it follows an anterograde secretory route which is triggered by short-term TSH stimulation of thyroid epithelial cells.

## 4. Materials and Methods

### 4.1. Cell Culture

Human thyroid epithelial cells (Nthy-ori 3-1) [16] were cultured in Roswell Park Memorial Institute medium (RPMI 1640, #12-702F, Lonza, Verviers, Belgium) supplemented with 10% fetal calf serum (#10270106, origin Brazil, Thermo Fisher Scientific, Darmstadt, Germany). Nthy-ori 3-1 cells were transduced to stably express eGFP-tagged full-length cathepsin V as described before [8], hereafter referred to as ‘Nthyori-CV’. Cell cultures were maintained at 37 °C in a humidified atmosphere at 5% CO_2_. It is important to note that metabolic activity, proliferation rates and morphological changes were not observed in Nthyori-CV when compared with Nthy-ori 3-1 cells (see Figure 2) and [8].

For stimulation, confluently grown Nthyori-CV cells were incubated in serum-free RPMI 1640 containing 100 μU/mL TSH (#869006, Merck, Darmstadt, Germany) for the indicated time intervals of up to 24 h.

### 4.2. Flow Cytometry Analysis

In order to quantify the cellular hCV-eGFP amounts upon TSH stimulation, cells were trypsinized and collected by centrifugation at 200× *g* for 5 min. After washing twice with phosphate-buffered saline (PBS) and resuspending the cells in one ml PBS, the green fluorescence intensity was quantified using a CyFlow Space flow cytometer (Sysmex, Dresden, Germany). Data was analyzed using FlowJo software (Tree Star, Ashland, OR, USA).

### 4.3. Indirect Immunofluorescence and Image Acquisition

Cells were paraformaldehyde-fixed, Triton X-100 permeabilized and immunolabeled as described before [8]. Immunohistochemistry was performed on paraffin-embedded human thyroid tissue sections obtained from patients undergoing thyroid surgery and used in compliance with the Helsinki Declaration [74]. The sections were deparaffinized and rehydrated by dipping them in xylol 4 times for 5 min each, followed by rinsing with decreasing concentrations of ethanol (100%, 95%, 70%, and 30%) for 5 min each. Slides were then incubated in freshly prepared sodium borohydride (1% in distilled water; Carl Roth GmbH, Karlsruhe, Germany) for 5 min at room temperature to reduce auto-fluorescence. After washing with calcium- and magnesium-free PBS (CMF-PBS), slides were blocked and immunolabeled as described before [8].

The following primary antibodies were used in this study: monoclonal mouse anti human cathepsin V (1:50; #MAB1080, R&D Systems, Minneapolis, MN, USA), monoclonal mouse anti human procathepsin V clone CV55-1C5 (1:100; produced by E.W.), polyclonal rabbit anti PDI (1:100; #ADI-SPA-890, Enzo Life Sciences, Lörrach, Germany), monoclonal mouse anti GM130 (1:100; #610822, BD Biosciences Laboratories, Allschwil, Switzerland), and polyclonal rabbit anti Lamp1 (1:100; #L1418, Merck, Darmstadt, Germany). As secondary antibodies, Alexa 546-conjugated goat anti mouse and goat anti rabbit IgG were used (1:200; #A11018, #A11071, respectively, Molecular Probes, Karlsruhe, Germany). Draq5™ (Bio-status Limited, Shepshed Leicestershire, UK) was used at a final concentration of 5 µM to counter-stain nuclear DNA.

Immunolabeled tissue sections and cells were mounted on microscope slides and imaged with a confocal laser scanning microscope equipped with Argon and Helium–Neon lasers (LSM 510 Meta; Carl Zeiss Jena GmbH, Jena, Germany). Micrographs were obtained at a pinhole setting of one Airy unit at resolutions of 1024 × 1024 pixels. Images were analyzed with the LSM 510 software, release 3.2 (Carl Zeiss Jena GmbH, Jena, Germany) and stored in TIFF format.

### 4.4. Subcellular Fractionation

All steps were performed on ice and centrifugations were done at 4 °C. Whole cell lysates were prepared by resuspending cells in lysis buffer consisting of 0.2% Triton X 100 in PBS, pH 7.4, supplemented with protease inhibitors, i.e., 10 µM E64, 1 µM Pepstatin A, 2 ng/mL Aprotinin, 0.02 M EDTA, as described before [8]. For the isolation of lysosome-enriched fractions, cells were washed three times with ice cold PBS, harvested with a cell scraper, and then collected by centrifugation at 200× *g* for 5 min. The pellets were resuspended in homogenization buffer containing 100 mM Soerensen phosphate buffer (KH_2_PO_4_ and Na_2_HPO_4_, pH 7.2), supplemented with 0.25 M sucrose and 5 mM EDTA. The cell suspensions were homogenized using a hand-held homogenizer for 3 min on ice. The homogenates were centrifuged at 900× *g* for 5 min to collect nuclei. The post-nuclear supernatants were then subjected to centrifugation at 10,000× *g* for 20 min to prepare lysosomes. The resulting pellets were washed twice with ice-cold PBS, resuspended in lysis buffer (see above), and incubated for 30 min at 4 °C on an end-over-end rotator. The lysates were cleared by centrifugation at 16,000× *g* for 10 min and supernatants were used as lysosome-enriched fractions. Protein concentrations in whole cell lysates and lysosomal fractions were determined by the Neuhoff assay [75].

### 4.5. TCA Protein Precipitation from Conditioned Media

Cells were grown in complete culture medium until confluence, then incubated in serum-free medium with or without TSH for 24 h. Conditioned media were collected and centrifuged at 900× *g* for 10 min at 4 °C to remove cell debris. In order to precipitate proteins, ice-cold trichloroacetic acid (TCA, Carl Roth GmbH, Karlsruhe, Germany) was added to the conditioned media at a final concentration of 10% (*v*/*v*). After incubation on ice for 1 h, samples were centrifuged at 13,000× *g* for 10 min at 4 °C. The supernatants were discarded, and pellets were dried by inverting the tubes on tissue paper for approximately 30 min at room temperature. The protein pellets were resuspended in SDS-PAGE sample buffer (10 mM Tris-HCl, pH 7.6, 0.5% SDS, 25 mM DTT, 10% glycerol and 25 μg/mL bromophenol blue). In order to adjust to neutral pH, 1.5 M Tris buffer (pH 8.8) was added dropwise, until the color of the samples changed from yellow to purple. Samples were normalized to equal protein concentrations based on the determined protein concentrations of the corresponding cell lysates as described previously [18]. The TCA-precipitated proteins were separated on SDS-PAGE gels as described below.

### 4.6. Detection of DCG-04-Labeled Cysteine Peptidases

The whole cell lysates were prepared by resuspending and incubating the cells in lysis buffer, i.e., 0.2% Triton X-100 in PBS, supplemented with 5 µM biotinylated DCG-04 [76] for 1 h at 4 °C on an end-over-end rotator. The supernatants were cleared by centrifugation at 16,000× *g* for 10 min at 4 °C. The Neuhoff assay [75] was used to determine protein concentrations. To isolate proteases covalently labeled with DCG-04, 250 µg of total protein per sample was incubated with 25 µL of Avidin-conjugated agarose beads for 1 h at 4 °C on an end-over-end rotator. The beads were collected by centrifugation at 200× *g* for 1 min at 4 °C and washed three times with ice-cold PBS. Afterwards, the beads were resuspended in 40 µL of SDS-PAGE sample buffer each, and heated at 95 °C for 5 min. The proteins covalently labeled by DCG-04 were separated on SDS-PAGE gels as described below.

### 4.7. Enzymatic de-Glycosylation of N-Linked Glycoproteins

Whole cell lysates of Nthyori-CV cells were prepared as described above. To cleave N-linked glycans, 100 µg of protein in a total volume of 27 µL each was treated with Endoglycosidase F1 or Peptide-N-Glycosidase F as described before [77]. Briefly, samples were incubated with denaturing buffer (final concentration 0.5% SDS and 40 mM DTT) at 95 °C for 10 min in order to linearize the proteins and increase their accessibility to the enzymes. Afterwards, EndoF1 or PNGase F digestion was performed in a reaction mixture containing 2% Triton X-100 in 50 mM sodium citrate, pH 5.5, or 50 mM sodium phosphate, pH 7.5, respectively. Enzymes were added to reach a final ratio of substrate:enzyme of 50:1 overnight at 37 °C. Samples were prepared for loading on SDS-PAGE gels by adding sample buffer and heating at 95 °C for 5 min.

### 4.8. SDS-PAGE and Immunoblotting

Protein samples were loaded onto 12.5% SDS-PAGE gels along with a Page Ruler Pre-stained Protein ladder (#26616, Thermo Scientific, Schwerte, Germany). Separated proteins were transferred onto nitrocellulose membranes using semi-dry Western blotting. Unspecific binding sites were blocked by incubating the membranes in 5% milk powder in PBS, supplemented with 0.3% Tween (PBS-T) overnight at 4 °C. Membranes were then incubated overnight at 4 °C with primary antibodies diluted in PBS-T, namely, mouse anti GFP (1:1500; #1814460, Roche, Mannheim, Germany), goat anti cathepsin B (1:1000; #GT15047, Neuromics, Hiddenhausen, Germany), goat anti cathepsin L (1:1000; #GT15049, Neuromics, Hiddenhausen, Germany), rabbit anti cathepsin D (1:80; #IM-16, Calbiochem through Merck, Darmstadt, Germany), mouse anti PCNA (1:1000; ab29, Abcam, Cambridge, UK), and rabbit anti β-tubulin (1:1000; #ab6046, Abcam, Cambridge, UK). The respective HRP-conjugated secondary antibodies (1:5000, Southern Biotech, Birmingham, AL, USA) were applied for 1 h at room temperature. After incubation with ECL horseradish peroxidase substrate (#34580, Thermo Fisher Scientific, Schwerte, Germany) for 3 min at room temperature, the blots were visualized through enhanced chemiluminescence onto XPosure™ films (Pierce via Thermo Fisher Scientific, Schwerte, Germany). Band densitometry analysis was performed using Image Studio Lite version 5.2 (LI-COR Biosciences GmbH, Bad Homburg, Germany).

### 4.9. Statistical Analysis

Data was analyzed by the use of GraphPad Prism 5.01 software (GraphPad, San Diego, CA, USA). Levels of statistical significance were determined by one-way ANOVA, followed by Tukey post hoc tests. Values of *p* < 0.05 were considered statistically significant.

## Abbreviations

Ca^2+^	free calcium
CD-MPR	cation-dependent mannose-6-phosphate receptor
CMF-PBS	calcium- and magnesium-free PBS
eGFP	enhanced green fluorescent protein
hCV-eGFP	human full-length cathepsin V tagged with eGFP
M6P	mannose-6 phosphate
Nthyori-CV	Nthy-ori 3-1 transduced with hCV-eGFP
Lamp1	lysosome-associated membrane protein 1
PBS	phosphate-buffered saline
PCNA	proliferating cell nuclear antigen
PDI	ER-resident protein disulfide isomerase
PLCβ	Gαq-phospholipase Cβ
rER	rough endoplasmic reticulum
RPMI	Roswell Park Memorial Institute medium
SCTP	*Stratum corneum* thiol protease
TH	thyroid hormone
Tg	thyroglobulin
TGN	trans-Golgi network
TSH	thyroid stimulating hormone
TSHR	TSH receptors
